# Reception to Dr. Jacobi

**Published:** 1910-06

**Authors:** 


### Reception to Dr. Jacobi

THE Medical Society of the State of New York, at its last meeting, it will be remembered, appointed a committee consisting of the former presidents and the president-elect of the society to arrange a testimonial to Dr. Jacobi in commemoration of his eightieth birthday.

The ceremonies took the form of a reception held at the New York Academy of Medicine, May 6, 1910, at which Dr. Joseph D. Bryant, chairman of the committee and a former president of the society, presided and made a short but befitting address on taking the chair. A bronze portrait in relief of the guest was presented to him, that an enduring memento of the occasion might ever be in Dr. Jacobi's possession.

Dr. Charles Jewett, reigning president of the state society in a few well chosen words presented the medallion, that was unveiled by Ruth McAneny, daughter of Burough President McAneny, and granddaughter of Dr. Jacobi.

In the presence of 800 friends, Dr. Jacobi responded, making one of his most felicitous little speeches,—a gift that he posseses in a rare degree. He was deeply affected by this action of the society and expressed his appreciation thereof in great tenderness. It was a rare occasion, conducted with dignity and simplicity, in honor of a grand man.

---

A NEW state law, says the *Buffalo Express,* makes it easier to punish frauds in the Regents' examinations. The offer to impersonate another in examinations, or to sell examination papers in advance, is now punishable by fine and imprisonment. Since much of importance depends, under the present system, upon the honesty of the examination, any stiffening of the law is well advised. If the examinations are to be held at all, their honesty should be above question.

In order to show that spitting on the sidewalks is dangerous to health,, an investigation has been made by Dr. John Robertson, Medical Health Officer of Birmingham, England, says the *St. Louis Medical Review,* which shows that 7 per cent. of the "spits" collected in public places contained consumption germs. On the other hand the dust collected from the floors of the cottages of the Adirondack Cottage Sanitarium has been found to be free of tuberculosis germs, showing that a careful consumptive is not dangerous.

---

President Taft sent to Congress April 9, 1910, a message which may result in important developments in the study of the causes of cancer. The President asks an appropriation of $50,000 for a laboratory in which to conduct investigations, and transmits the recommendations of the Secretary of Commerce and Labor, the Commissioner of Fisheries, and Dr. H. R. Gaylord, director of the New York State Cancer Laboratory, for an inquiry into the cause of cancer in fishes.

"The very great importance of pursuing the investigation into the cause of cancer," said the President, "cannot be brought home to the Congress or to the public more acutely than by inviting attention to the memorandum of Dr. Gaylord herewith. Progress in the prevention and treatment of human diseases has been marvelously aided by an investigation into the same disease in those of the lower animals which are subject to it, and we have every reason to believe that a close investigation into the subject of cancer in fishes, which are frequently swept away by an epidemic of it, may give us light upon this dreadful human scourge." The President, during his recent visit to Buffalo, visited the State cancer laboratory to inspect the plant and the work there being done.

---

Dr. E. G. Martin, recently lecturing at the Harvard Medical School on "How to Breathe," disposed or attempted to dispose of the commonly accepted idea that to breathe deeply means a better and more complete supply of oxygen to the body. "There is no reason," said Dr. Martin, "why a healthy person should breathe deeply. Shallow breathing supplies all the oxygen the blood cells are able to carry to the tissues of the body. There is a distinct drawback to breathing deeply, for the more you ventilate the lungs the greater is the elimination of the carbon dioxide. This removes the stimulation from the brain center which helps the normal control of respiration."

King Edward was a friend of the medical profession, a supporter with influence and money of philanthropic enterprises, a promoter of medical progress and enlightenment. Attached to the royal household were twenty-five medical men, physicians and surgeons, all men of the highest skill and reputation. Among them Sir Francis Laking was perhaps the most frequently called upon for advice by his majesty. The king bestowed titular honors on members of the medical profession with liberality, and his death will be mourned by physicians all over the world who appreciated his interest in medical science and its advancement.

